# Comparison of different methods for DNA-free RNA isolation from SK-N-MC neuroblastoma

**DOI:** 10.1186/1756-0500-4-3

**Published:** 2011-01-06

**Authors:** Lucélia Tavares, Paula M Alves, Ricardo B Ferreira, Claudia N Santos

**Affiliations:** 1 Disease & Stress Biology, Instituto de Tecnologia Química e Biológica, Universidade Nova de Lisboa, 2781-901 Oeiras, Portugal; 2 Animal Cell Technology Unit, Instituto de Tecnologia Química e Biológica, Universidade Nova de Lisboa/Instituto de Biologia Experimental e Tecnológica, 2781-901 Oeiras, Portugal; 3 Instituto Superior de Agronomia, Centro de Botânica Aplicada à Agricultura, Universidade Técnica de Lisboa, Tapada da Ajuda, 1349-017 Lisboa, Portugal

## Abstract

**Background:**

RNA quality and quantity are important factors for ensuring the accuracy of gene expression analysis and other RNA-based downstream applications. Extraction of high quality nucleic acids is difficult from neuronal cells and brain tissues as they are particularly rich in lipids. In addition, most common RNA extraction methods are phenol-based, resulting in RNA that may be incompatible with downstream applications such as gene expression.

**Findings:**

In this work, a comparative analysis of the RNA quality obtained from SK-N-MC cells was performed using six commonly used RNA isolation kits: two phenol-based kits and four non-phenol based kits. The non-phenol based kits tested AxyPrep Multisource Total RNA Miniprep, RNeasy^® ^Mini, EasySpin and Ilustra RNAspin Mini RNA Isolation, all performed well and resulted in the isolation of high quality RNA, as evaluated by A_260_/A_280_. The RNA extracted with AxyPrep Multisource Total RNA Miniprep, RNeasy^® ^Mini and EasySpin provided the highest RNA yields. In particular, the RNA isolated by AxyPrep Multisource Total RNA Miniprep Kit did not show any detectable genomic DNA contamination even without previous DNase treatment or after RNA direct PCR amplification using universal 18S primers.

**Conclusions:**

The RNA extracted from SK-N-MC cells with AxyPrep Multisource Total RNA Miniprep Kit was superior with respect to the RNA quality and concentration. This kit does not use aggressive organic solvents and RNA free of genomic DNA was isolated without the need for DNase treatment.

## Background

The accuracy of gene expression evaluation is influenced by the concentration and quality of input RNA. The purity and integrity of RNA are critical elements for the overall success of RNA-based analyses [1]. Starting with a low quality RNA may compromise the results of downstream applications which are often labour-intensive, time-consuming and very expensive [2,3]. The integrity of the total RNA used should be examined prior to its use in quantitative RT-PCR, microarrays and any array-based applications.

To ensure acceptable total RNA quality, the RNA extraction procedure must fulfill a number of requirements: including, the final preparation must be free from protein, genomic DNA or enzyme inhibitors and must not include any phenol or alcohol carryover which may compromise downstream reactions [4]. Also, the purified RNA should also be free of nucleases to maintain integrity under appropriate storage conditions. Reverse transcriptase and PCR reactions are strongly dependent on the purification and clean-up methods, as well as on the presence of exogenous contaminants. For example, the presence of hemoglobin, fat, glycogen, Ca^2+^, high genomic DNA concentrations, DNA binding proteins or other cell constituents are critical contaminants [5,6].

There are three major techniques extensively used for RNA extraction: organic extraction, such as phenol-Guanidine Isothiocyanate (GITC)-based solutions, silica-membrane based spin column technology, and paramagnetic particle technology. One of the most commonly used methods is the phenol-GITC-based organic extraction. However, RNA samples isolated by this method are frequently contaminated with proteins and other cellular materials, organic solvents such as phenol-chloroform, salts and ethanol. Additionally, these methods require safety precautions (i.e., the use of fume hoods) which lengthen the procedure and employ liquid-liquid extraction leading to incomplete phase separation and increased carryover contamination with genomic DNA. Silica column and paramagnetic particle based RNA isolation systems do not require the use of toxic organic solvents, are relatively simple, efficient, low cost, and yield total intact RNA with low levels of contamination from proteins and other cellular materials [7]. However, these methods can often result in significant levels of genomic DNA contamination.

Digestion with DNase removes traces of DNA and is compulsory if the RNA samples are destined for use in RT-qPCR. DNase digestion after the final RNA precipitation step involves adding extra salts and proteins to the sample and since this can affect the efficiency of the cDNA synthesis, additional purification steps are required.

In this work, a comparative analysis of the RNA quality achieved from a neuroblastoma cell line (SK-N-MC) by six commonly used RNA isolation kits is presented; two phenol-based kits and four kits utilizing non-aggressive solvents. For the SK-N-MC cell line in particular, both types of extraction methods have previously been described, but RNA has been isolated mainly using phenol-GITC-based methods [8-13].

## Results

RNA isolation methods such as acid phenol extraction, glass fibre filter purification, and single-step reagents can provide RNA with acceptable quality. However, all RNA isolation methods do not have the ability to completely remove genomic DNA contamination from RNA samples. To evaluate the differential efficiency in obtaining RNA with minimal DNA contamination, six commercial kits for RNA extraction were tested using SK-N-MC cells. Among these kits, two of them (TRIzol^® ^Plus RNA Purification System (Invitrogen) and E.Z.N.A.™ Total RNA kit II (Omega Bio-Tek)) involve a more aggressive methodology which includes a mono-phasic solution of phenol and guanidine isothiocyanate. E.Z.N.A.™ Total RNA Kit II was selected for this analysis because it is mainly designed for fatty tissues by combining the advantage of one step RNA isolation technology and silica-membrane technology.

Among the six kits tested, AxyPrep Multisource Total RNA Miniprep, RNeasy^® ^Mini, EasySpin and Illustra RNAspin Mini RNA Isolation allow the isolation of higher quality RNA when compared to the other two kits (Table 1). The kits from Axygen, Qiagen, Citomed and GE, respectively, were qualitatively superior, providing a good A_260_/A_280 _ratio (around 2.10). An A_260_/A_280 _ratio greater than 1.8 is usually considered an acceptable indicator of good quality RNA with a low level of protein contamination [14,15]. An A_260_/A_230 _ratio higher than 1.8 is used as an indicator of extracted RNA with a low level of polysaccharides contamination. Quantitatively, the highest RNA concentration and yield was obtained by the AxyPrep Multisource Total RNA Miniprep kit while RNeasy^® ^Mini kit, EasySpin kit and TRIzol^® ^Plus RNA Purification System presented intermediate values, and Illustra RNAspin Mini RNA Isolation kit and E.Z.N.A.™ Total RNA kit II provided the lowest recovery values (Table 1). However, based on the standard deviation illustrated in Table 1, the TRIzol^® ^Plus RNA Purification System -(GITC based procedure) demonstrates low RNA recovery reproducibility. In terms of yield, among Axygen, Qiagen and Citomed kits, clearly the highest and most reproducible is the AxyPrep Multisource Total RNA Miniprep Kit, where RNA yield is at least four fold higher than the other methods. E.Z.N.A.™ Total RNA Kit II, a GITC based kit, and Illustra RNAspin Mini RNA Isolation Kit revealed rather poor efficiencies of RNA extraction from this neuroblastoma cell line (Table 1).

**Table 1:** Evaluation of quality and quantity parameters of RNA samples extracted from SK-N-MC neuroblastoma.

Kit	A_230_	A_260_	A_280_	A_260_/A_280_	A_260_/A_230_	Concentration (ng/μL)	Elution volume (μL)	Yield (μg RNA/1E6 cells)
AxyPrep Multisource Total RNA Miniprep Kit (Axygen)	5.38	6.82	3.29	2.07	1.26	272.80 ± 28.55	100	3.94 ± 0.41

RNeasy^® ^Mini Kit (Qiagen)	1.32	2.14	1.03	2.07	1.63	85.74 ± 72.26	50	0.62 ± 0.52

EasySpin (Citomed)	1.79	3.54	1.73	2.06	1.92	141.5 ± 74.58	50	1.02 ± 0.54

Illustra RNAspin Mini RNA Isolation Kit (GE)	0.3	0.15	0.07	2.13	0.37	5.98 ± 7.44	100	0.09 ± 0.11

TRIzol^® ^Plus RNA Purification System (Invitrogen)	1.13	2.31	1.15	1.86	1.65	92.4 ± 111.50	50	0.67 ± 0.80

E.Z.N.A. ™ Total RNA Kit II (Omega bio-tek)	0.30	0.55	0.29	1.89	1.52	21.87 ± 4.14	50	0.16 ± 0.03

Most gene expression experiments require RNA samples free of DNA contamination, therefore it is imperative to minimize this contamination. Removal of DNA is especially critical for RT-PCR applications, since DNA can be amplified during the PCR portion of the experiment, resulting in false positive results and high background "noise" levels.

**Figure 1:**
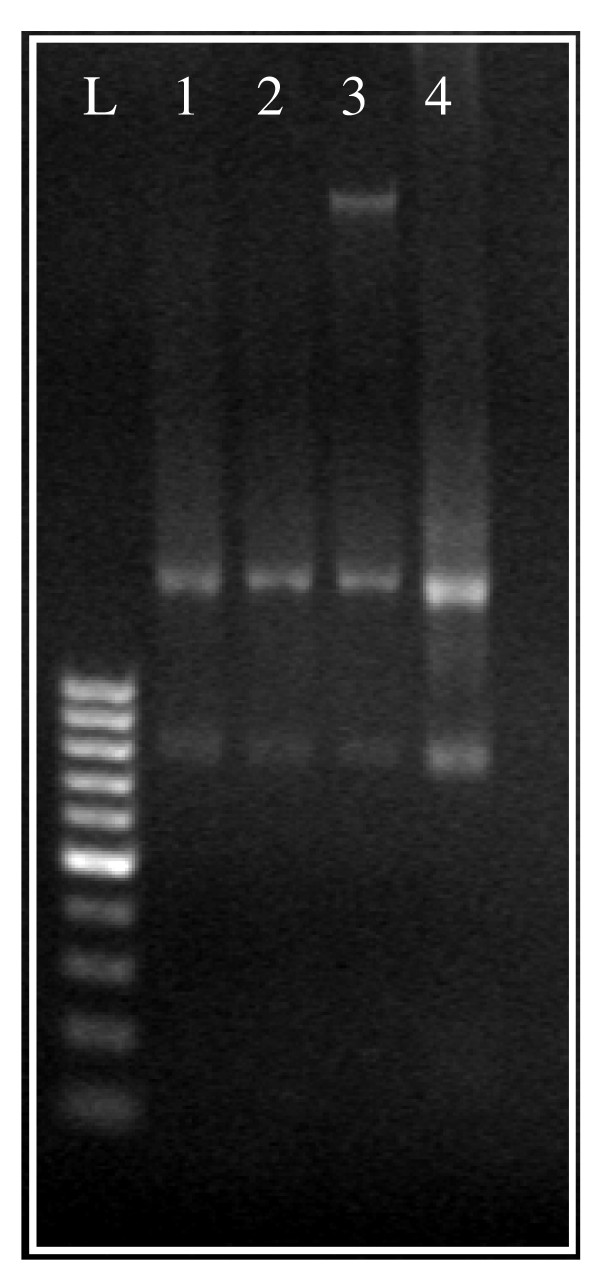
**Electrophoresis of RNA samples in 2% (w/v) agarose gel, stained with ethidium bromide**. L- 100 bp ladder; 1- RNA (untreated sample) isolated by AxyPrep Multisource Total RNA Miniprep kit (Axygen); 2- RNA (treated with Turbo™ DNase, Ambion) isolated by AxyPrep Multisource Total RNA Miniprep kit (Axygen); 3- RNA (DNase I untreated sample) isolated by RNeasy^® ^Mini kit (Qiagen); 4- RNA (treated with Turbo™ DNase, Ambion) isolated by RNeasy^® ^Mini kit (Qiagen).

**Figure 2:**
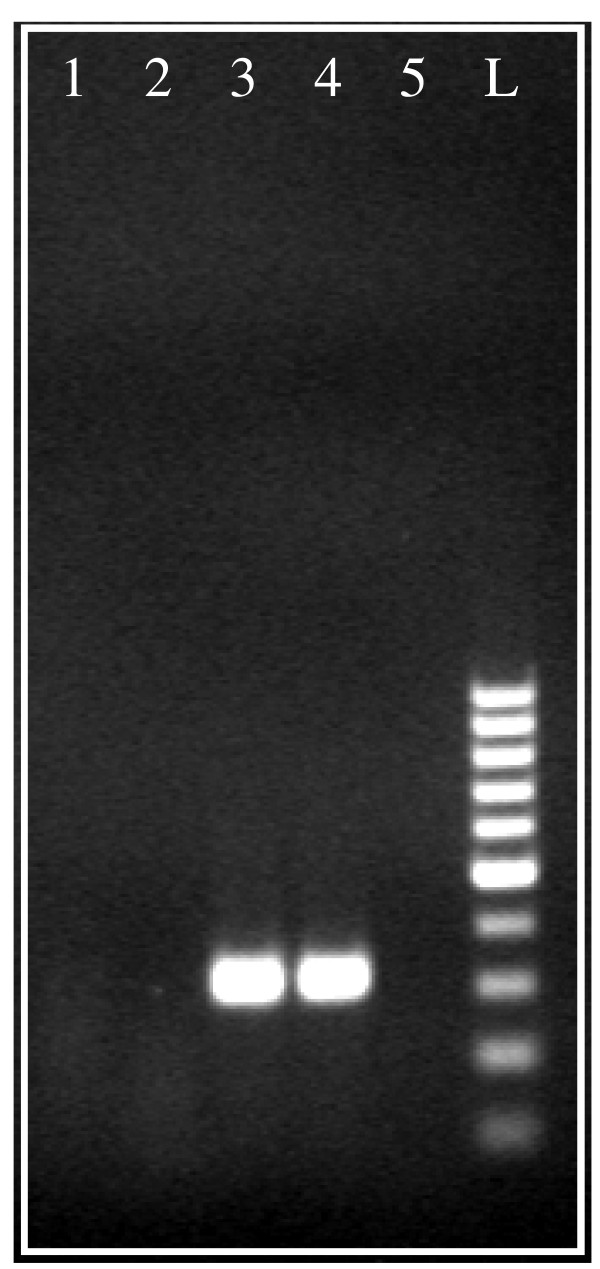
**Electrophoresis of 18S PCR amplification products of RNA samples without reverse transcription, in 2% (w/v) agarose gel, stained with ethidium bromide**. 18S PCR amplification products from RNA isolated by AxyPrep Multisource Total RNA Miniprep Kit (Axygen); 2-18S PCR amplification from RNA isolated by AxyPrep Multisource Total RNA Miniprep Kit (Axygen) and treated with Turbo™ DNase (Ambion); 3-18S PCR amplification products from RNA isolated by RNeasy^® ^Mini Kit (Qiagen); 4-18S PCR amplification products from RNA isolated by RNeasy^® ^Mini Kit (Qiagen) and treated with Turbo™ DNase (Ambion); 5-18S PCR amplification products from water (negative control); L- 100 bp ladder.

DNase I digestion has consistently proven to be the most effective method for removing DNA contamination from RNA samples. DNase I treatment efficacy test was evaluated for the two best performing kits; RNA extracted with AxyPrep Multisource, Total RNA Miniprep and RNeasy^® ^Mini. Results from kits, with or without DNase treatment, were visualized in agarose gel stained with ethidium bromide (Figure 1). RNA isolated with AxyPrep Multisource Total RNA Miniprep kit did not show visible genomic DNA contamination even without DNase treatment (Figure 1, lanes 1 and 2). On the contrary, RNA extracted using the RNeasy^® ^Mini Kit clearly contained DNA contamination, which disappeared promptly after DNase treatment. Very low levels of DNA contamination, albeit not detectable by agarose gel electrophoresis, may be amplified and then corrupt the results obtained by highly sensitive techniques such as Real-Time PCR. In an attempt to address and evaluate this hypothesis, RNA samples (with and without DNase treatment) isolated by Axygen and Qiagen kits were directly amplified by PCR using universal 18S primers. The corresponding reaction products where visualized by agarose gel electrophoresis (Figure 2). Although not visualized by direct RNA electrophoresis in Figure 1, RNA isolated using the RNeasy^® ^Mini kit and treated with DNase was shown to contain DNA contamination after PCR amplification (Figure 2, lanes 3 and 4). This result was obtained after confirming experimentally that the PCR product amplification was a result of the RNA concentration in the sample (and therefore the DNA contaminant amount) and was not due to extensive amplification (results not shown). After direct PCR amplification, the RNA isolated by AxyPrep Multisource Total RNA Miniprep kit (with or without previous DNase treatment) did not reveal the presence of any band on the agarose gel (Figure 2, lanes 1 and 2). Therefore, the Axygen kit provides reliable and good quality RNA isolation from SK-N-MC neuroblastoma cells, thus suitable for a successful RNA amplification without the need of any DNase treatment. DNase treatment is considered disadvantageous by some investigators, as it adds extra salts and protein to the sample and can affect the efficiency of the subsequent cDNA synthesis.

## Conclusions

Quality control is an extremely important issue when isolating RNA, especially when the quantity is small and the amount required is large, for example microarray experiments (15 μg). In regards to quality and yield the AxyPrep Multisource Total RNA Miniprep kit was determined to be the best kit tested for the isolation of RNA from SK-N-MC cells. This kit uses no aggressive organic solvents and delivers RNA devoid of genomic DNA, without the need for DNase treatment. This is an important finding, especially in large scale gene-expression studies, since DNase treatment is time consuming and adds a substantial cost to the overall cost for such experiments. Furthermore, DNase treatment may lead to a loss in both RNA amount and mRNA integrity due to the exposure of the RNA samples to high temperatures during the heat inactivation step required for many commercial DNases. This effect extends further to include any downstream applications demanding compulsory genomic DNA removal. AxyPrep Multisource Total RNA Miniprep Kit allows the use of mild treatments minimizing the introduction of further contaminants to the extracted RNA.

## Methods

### Cell culture

Human neuroblastoma SK-N-MC cells were obtained from the European Collection of Cell Cultures (ECACC) and were cultured in EMEM supplemented with 2 mM glutamine, 10% (v/v) heat-inactivated fetal bovine serum (Gibco), 1% (v/v) of non-essential amino acids (Sigma) and 1 mM sodium pyruvate. Cells were cultivated at 37°C in a humidified atmosphere containing 5% (v/v) CO_2_. For routine culture, cells were grown until reaching approximately 90% confluence. For RNA isolation, cells were harvested using trypsin and stored frozen at -80°C.

### RNA isolation

For SK-N-MC cell RNA isolation, six commercially available kits were tested: AxyPrep Multisource Total RNA Miniprep (Axygen), RNeasy^® ^Mini (Qiagen), EasySpin (Citomed), Illustra RNAspin Mini RNA Isolation (GE), TRIzol^® ^Plus RNA Purification System (Invitrogen) and E.Z.N.A.™ Total RNA kit II (Omega Bio-Tek). The same amount of cells (6.92E6) and the manufacturer protocols were followed for each kit.

When appropriate, the isolated RNA was treated with Turbo™ DNase I (Ambion), accordingly to the manufacturer's instructions.

For assessing RNA quality and yield, A_260/A280 _and A_260/A230 _ratios for RNA preparation samples were analysed with a Nano-Drop^® ^ND-1000 spectrophotometer (NanoDrop Technologies). RNA integrity and DNA contamination were determined by 28S/18S rRNA visualization in agarose gel, stained with ethidium bromide.

PCR was used to detect potential DNA contamination, using primers specific for 18S rRNA as Quantum RNA Universal 18S Internal Standards primers (Ambion). PCR reaction contained 1 μL of input (20-200 ng RNA), 0.6 U Taq DNA Polymerase (Fermentas), 2.5 μL of 18S PCR Primer Pair (Ambion), 2 mM MgCl_2_, 0.2 mM of each dNTP (Invitrogen), 75 mM Tris-HCl (pH 8.8 at 25°C), 20 mM (NH_4_)_2_SO_4 _and 0.01% (v/v) Tween 20. The following program was applied: 1 cycle of 3 min. at 95°C for denaturation, followed by 30 cycles (30 s at 95°C for denaturation, 30 s at 57°C for annealing, 30 s at 72°C for extension) and a final 5 min extension at 72°C.

PCR products were visualized in agarose gels, stained with ethidium bromide.

## Competing interests

The authors declare that they have no competing interests.

## Authors' contributions

LT performed the experiments. All authors contributed to the conception, design, analysis, and interpretation of the data, drafting of the manuscript and revision for important intellectual content. All authors read and approved the final version of the manuscript.
